# Predominant Intractable Nausea in the Diagnosis of Bulbar Myasthenia Gravis: A Case Study and Review of Literature

**DOI:** 10.7759/cureus.32553

**Published:** 2022-12-15

**Authors:** Tripti Nagar, Jahanavi M Ramakrishna, Vatsal Khanna, Issam Turk

**Affiliations:** 1 Internal Medicine, Wayne State University School of Medicine, Rochester Hills, USA; 2 Internal Medicine, Wayne State University, Rochester Hills, USA; 3 Department of Gastroenterology, Ascension Providence Rochester Hospital, Rochester Hills, USA

**Keywords:** intractable nausea, total perenteral nutrition, health related quality of life myasthenia gravis, gastrointestinal motility, adult gastroenterology

## Abstract

Gastrointestinal (GI)-predominant myasthenia gravis (MG) is rare and presents a complex clinical scenario. We report the case of a 73-year-old female with dysphagia and intractable nausea found to have bulbar MG. Her symptoms persisted despite conventional MG management with plasma exchange therapy and anticholinergics. We review existing literature and discuss the clinical manifestations, diagnosis, and treatment of bulbar MG. This case highlights the need for novel MG treatment modalities in patients like ours with anomalous, GI-predominant MG who might not respond to conventional management.

## Introduction

Myasthenia gravis (MG) is a commonly diagnosed neurologic condition that targets the acetylcholinesterase receptors of the neuromuscular junction. The incidence rate of MG is approximately 2-4 million cases per year [1]. However, gastrointestinal (GI) signs and symptoms are a rare manifestation of MG with limited data on GI-predominant presentations. Atypical presentations are more commonly seen in an elderly population, leading to diagnostic delays and increased severity of presentation.

Literature on how to approach MG patients with GI disease components is lacking. The few reports published focus primarily on dysphagia and note symptom resolution with pharmacologic treatment of MG [1,2]. We present a case of a 73-year-old female who presented with intractable nausea that led to a new diagnosis of bulbar MG. This case represents the importance of broad differentials encompassing neurogastro motility etiologies in the evaluation of nausea and poses further questions regarding the long-term management of patients who do not achieve symptomatic relief.

## Case presentation

A 73-year-old female presented to our hospital with complaints of dysphagia and nausea beginning three weeks prior. She described waxing and waning difficulty swallowing solids and liquids associated with globus sensation. Her nausea was unrelenting and persisted even when her dysphagia was absent. 

Physical examination revealed slight drooping of the left side of her lip but was otherwise unremarkable. The patient endorsed that the left facial droop was consistent with her baseline; the National Institutes of Health (NIH) stroke scale score was zero. Computed tomography and magnetic resonance imaging of the head and neck ruled out acute cerebrovascular disease, hemorrhage, or laryngeal nerve injury. For immediate symptomatic relief of her nausea, the patient was started on intravenous ondansetron. Evaluation by speech therapy showed no abnormalities with swallow physiology. A barium swallow study was recommended, but the patient’s symptoms were so severe that she was unable to tolerate the exam. It was decided to pursue upper endoscopy, however, this study was also negative with no anatomical abnormalities of the esophagus. 

Throughout the patient’s hospital course, her nausea and dysphagia remained despite trying multiple antiemetic agents, including ondansetron, trimethobenzamide, and prochlorperazine. Despite optimized antiemetic therapy, the patient continued to experience nausea and decreased appetite. At this point, she was unable to tolerate oral intake and refused supplemental nutritional therapy, including peripheral or total parenteral nutrition (TPN). As organic GI causes of dysphagia and nausea had been effectively ruled out; an MG reflex panel was ordered to evaluate for neuromuscular etiologies of her symptomatology. The reflex panel was positive for acetylcholine receptor blocking and binding antibodies. Post-diagnostic review of the patient was suspicious for ongoing episodic musculoskeletal symptoms that would resolve spontaneously over the past six months. She felt her speech was “garbled” and had an episode of difficulty holding her head up. Physical examination was significant for fatigable ptosis more significant on the left, extended fatigue and recovery with sustained upward gaze of 120 seconds. Large muscle fatigue was evident in the upper and lower extremities demonstrated by the inability to hold her arms in abduction for longer than 15 seconds, she was also unable to elevate her legs while in bed for longer than 15 seconds. Bulbar muscle fatigue produced dysarthria when the patient was instructed to count from 1-50 and dysphonia due to laryngeal muscle fatigue while asked to produce a high-pitched sound. Given the severity of her symptoms, plasma exchange therapy was initiated. She underwent five rounds over the next 10 days providing rapid symptomatic improvement and allowing the patient to tolerate a low-volume liquid oral diet. She was started on long-term maintenance therapy with anticholinergic medication. 

Despite optimal treatment and transient clinical improvement, the patient continued to struggle with debilitating nausea. She continued to deteriorate despite optimized medical management. She eventually decided to pursue comfort care measures and passed away due to severe electrolyte abnormalities and resulting cardiovascular complications.

## Discussion

GI manifestations of MG are rare in initial disease presentation and make the diagnosis challenging to reach. Oftentimes, MG patients who have GI complaints first present with ocular MG that then progress to bulbar symptom development and large muscle fatigability [1]. There is limited data regarding the incidence of bulbar symptoms in MG. One study reported that only 6% of MG patients endorsed bulbar symptoms on initial presentation and approximately 15% list dysphagia as the sole complaint [2,3]. Furthermore, a large percentage (30%-60%) describe experiencing dysphagia at some point in their disease course [4]. Atypical GI presentations, particularly with the absence of other neuromuscular manifestations, can be missed and autoimmune disorders should be included in the differential. 

Dysphagia in MG stems from the primary involvement of bulbar and laryngeal muscles, and the secondary involvement of striated muscles of the head, neck, and esophagus [5,6]. Despite studies on bulbar/laryngeal subsets of MG, there is insufficient literature on the mechanism of unresolving nausea. Our literature review showed no prior case report describing intractable and unresolved nausea despite optimal MG treatment. MG has been implicated in causing intestinal pseudo-obstructions presenting as nausea and vomiting. However, this is seen as a classic presentation of small bowel obstruction, rather than an initial manifestation of MG [7]. One plausible explanation is the development of autonomic instability secondary to MG that leads to potential widespread neuropathy [8]. This can affect the tone of the vagus nerve, causing a pathophysiologic phenomenon similar to gastroparesis. Autonomic sequelae are seen with high autoantibody burden and are associated with poor outcomes as well as increased incidence of myasthenic crisis [9].

Symptomatology of autonomic involvement varies and can include multiple organ systems, including gastric and intestinal dysmotility. A case series by Vernino et al. examined seven cases of MG with associated dysautonomia. Five of seven patients had GI complaints and their symptoms drastically improved or resolved with adequate treatment of MG with anticholinesterase therapy [10]. Due to low initial suspicion for autonomic dysregulation in our patient, a gastric emptying study was not done. Early diagnosis of atypical MG is directly correlated to early treatment and avoidance of complications, including but not limited to aspiration pneumonia, malnutritive conditions, and traumatic esophageal lesions.

While autonomic dysfunction has been reported in a few patients with MG, those with GI symptoms improve drastically after initiating anticholinesterase therapy. More severe presentations, such as the one seen above, are first treated with plasma exchange therapy to decrease the autoantibody burden prior to initiation of anticholinergic pharmacotherapy. These treatment methods, however, are not without concern as anticholinergic medications have well-known side effect profiles significant for nausea. Judicious clinical discernment is required to ensure an adequate diagnosis is achieved and appropriate therapies are not exacerbating baseline complaints instead of providing therapeutic solutions. Standard therapies for gastroparesis, including prokinetic agents, can be attempted for symptom resolution. However, erythromycin is avoided with caution as some cases have demonstrated effects ranging from aggravation of MG to induction of myasthenic crisis [11]. Initiation of TPN in the setting of refractory GI MG has not yet been addressed in the literature. However, this case poses an interesting question of whether supplementation of nutritional status has the potential of leading to clinical improvement.

## Conclusions

Anomalous, GI-predominant presentations of MG pose a challenge to clinicians. With an extraordinarily wide differential for nausea and dysphagia, neuromuscular etiologies such as MG are not prioritized differential diagnoses. Future research should focus on bulbar MG management, including the role of supplemental nutrition and novel modalities for refractory GI complaints.
